# Primary Necrosis of the Ligamentum Teres Hepatis: A Rare Cause of Acute Abdominal Pain

**DOI:** 10.5334/jbsr.3232

**Published:** 2023-07-21

**Authors:** Rui Yang, Ying Zhao, Qingyu Ji

**Affiliations:** 1 The Second Affiliated Hospital of Baotou Medical College, Inner Mongolia University of Science and Technology, China

**Keywords:** ligamentum teres hepatis, primary necrosis, CT, acute abdominal pain

## Abstract

Primary necrosis of the ligamentum teres hepatis (PNLTH) is an extremely rare disease which presents with acute abdominal pain. It has easily been misdiagnosed due to lack of clinical manifestations. Early recognition of PNLTH is crucial for treatment while CT is a good methed to diagnose and differential diagnose PNLTH.

**Teaching Point:** Primary necrosis of the ligamentum teres hepatis (PNLTH) is an extremely rare disease characterized with acute abdominal pain, while computed tomography is the recommended technique to diagnose and differential diagnose this disease.

## Case History

**Figure 1:**
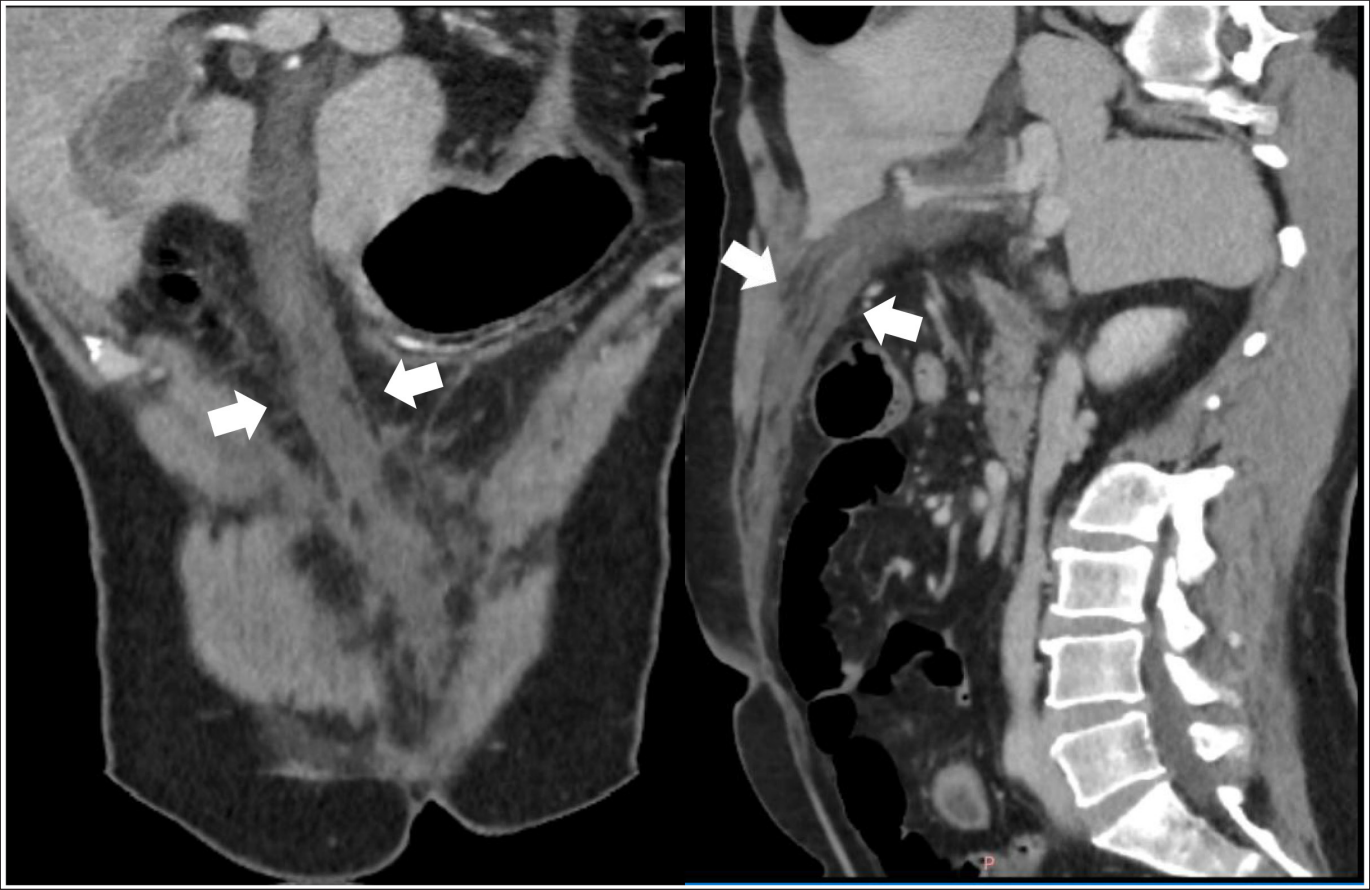


**Figure 2:**
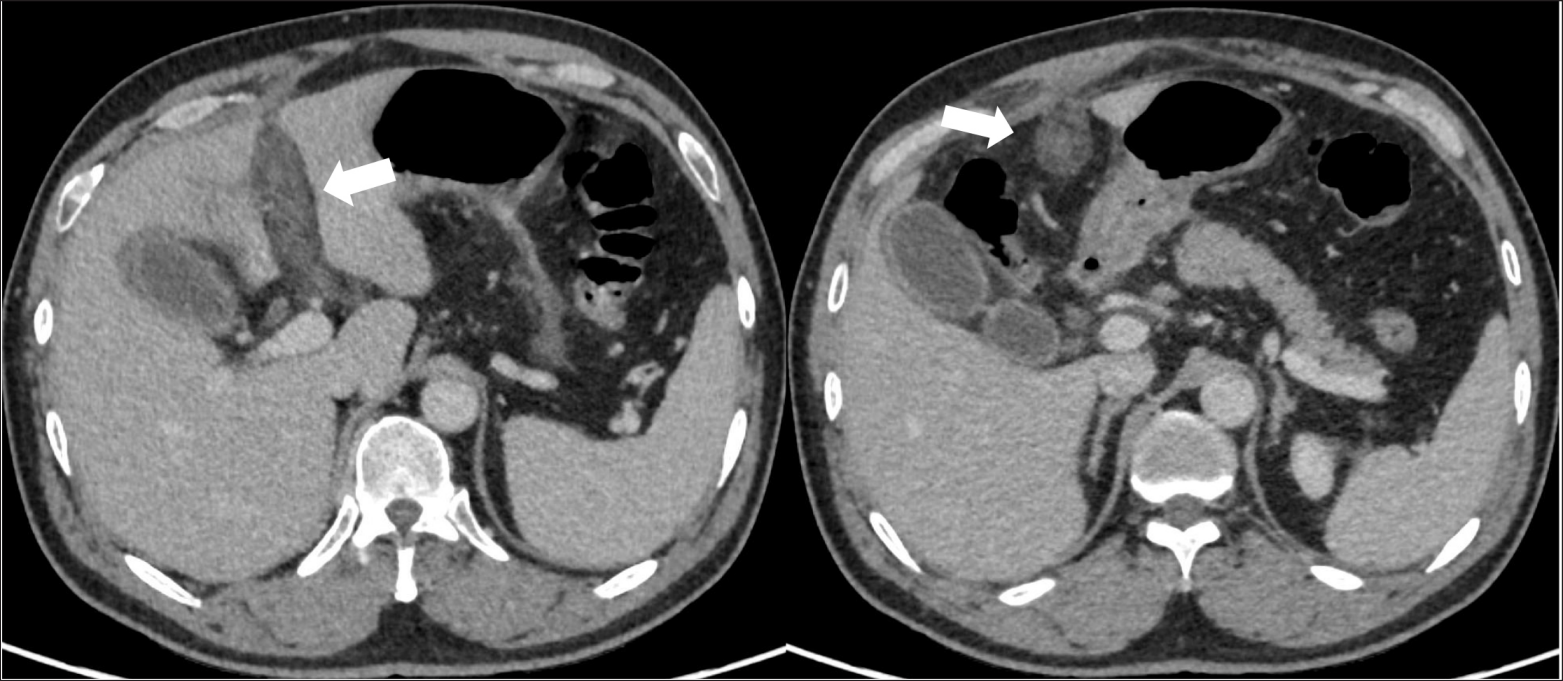


A 48-year-old male coal miner presented to our hospital with persistent pain in the upper and middle abdomen accompanied with fever. Physical examination showed epigastric muscle tension with significant pressure pain mainly under the glabella, and no mass was palpable. He had a history of chronic gastritis. Significant laboratory tests included serum amylase 621 U/L (normal value 17–115 U/L) and WBC 21 × 10^9^/L (normal value 4–10 × 10^9^/L). Liver function tests with normal enzymatic profile and bilirubin was plain. The patient was suspected with acute pancreatitis and subsequently received an enhanced computed tomography (CT) scan for further diagnosis. The CT examination of the abdomen showed normal morphology of pancreatic with fewer exudation above the body of pancreas. A hypodensity strip (Figure 1, white arrow) with no significant enhancement (Figure 2, white arrow) was found beside the left branch of the portal vein. It extended along the round ligament and ended at the navel.

**Figure 3:**
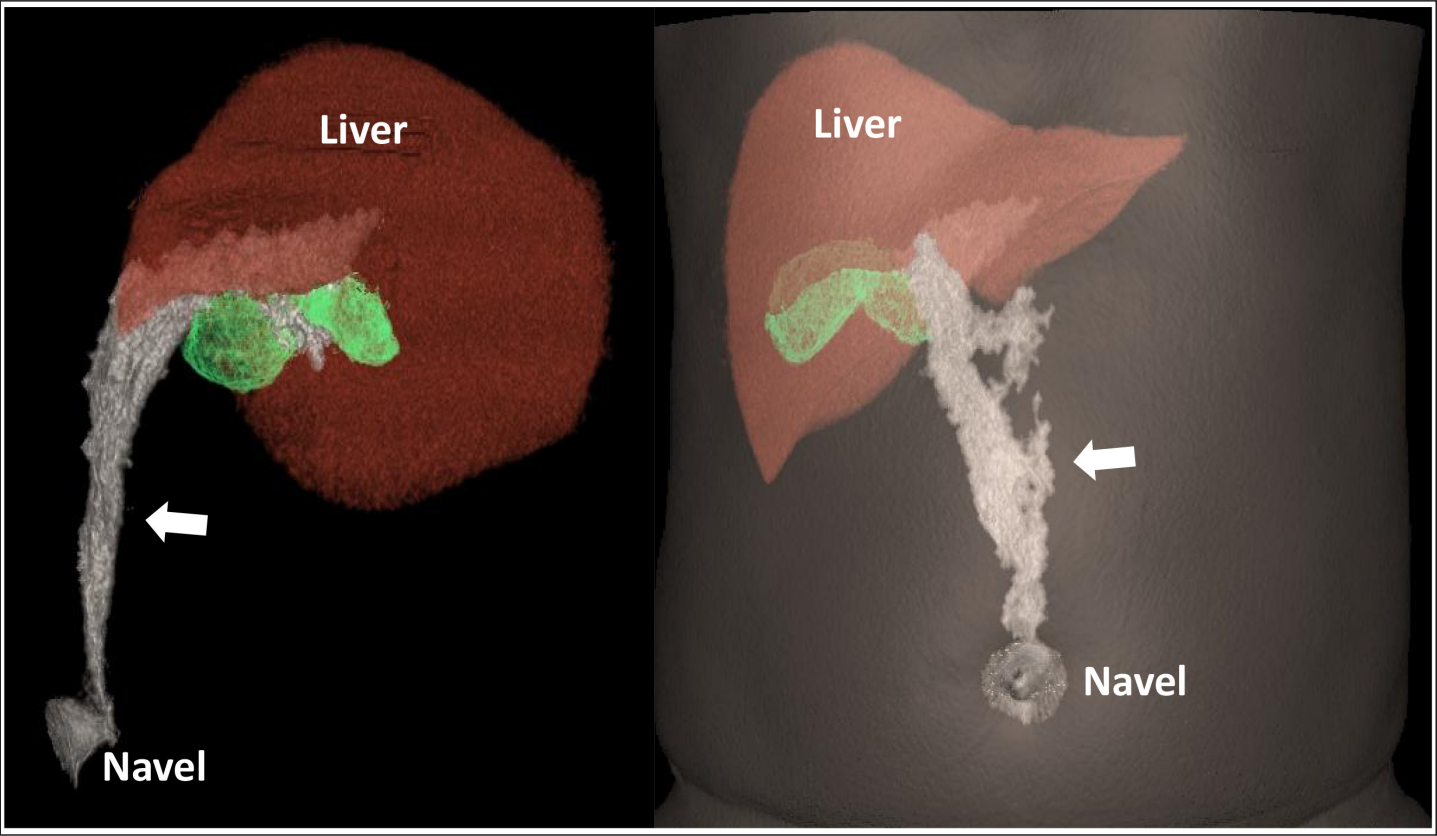


CT results suggested the diagnosis of necrosis of the ligamentum teres hepatis and provided a CT volume reproduction of the lesion to show its neighboring relationships (Figure 3, white arrow). To avoid serious complications, the clinician removed the lesion with minimally invasive surgery via laparoscopy and pathology results confirmed the diagnose of PNLTH.

## Comments

The hepatic round ligament is a remnant of umbilical vein atresia, formed by the embryonic remnants of degenerative atresia of the left umbilical vein, which connects to the left hepatic vein or inferior vena cava through the venous ligament. The clinical incidence of PNLTH is extremely low and the pathogenesis is unclear [1]. In this case, the patient was a coal miner with poor navel hygiene, so we speculate that perhaps a retrograde infection through the navel caused the PNLTH in this patient. The patient presented with elevated serum amylase, and we considered that was because the necrotic material stimulated margins of the pancreas and induced a mild inflammatory response of the pancreas [1]. Through the case, we know PNLTH can present with abnormal blood amylase. For patients suspected with acute pancreatitis, PNLTH should be taken into diagnosis and differential diagnosis. An enhance CT examination is recommended to clarify the diagnosis. In addition, the use of CT volumetric reproduction techniques can visualize the source and extent of the lesion and provide clinical assistance.
